# A Structural Bioinformatics-Guided Study of Adenosine Triphosphate-Binding Cassette (ABC) Transporters and Their Substrates

**DOI:** 10.3390/membranes15010020

**Published:** 2025-01-10

**Authors:** Iqra Younus, Robert C. Ford, Stephen M. Prince

**Affiliations:** Faculty of Biology, Medicine and Health, School of Biological Sciences, The University of Manchester, Manchester M13 9PT, UK; robert.ford@manchester.ac.uk (R.C.F.); steve.prince@manchester.ac.uk (S.M.P.)

**Keywords:** ABC transporter, ortholog, *Saccharomyces cerevisiae*, bioinformatics, membrane protein

## Abstract

Adenosine triphosphate-binding cassette (ABC) transporters form a ubiquitous superfamily of integral membrane proteins involved in the translocation of substrates across membranes. Human ABC transporters are closely linked to the pathogenesis of diseases such as cancer, metabolic diseases, and Alzheimer’s disease. In this study, four ABC transporters were chosen based on (I) their importance in humans and (II) their score in a structural bioinformatics screen aimed at the prediction of crystallisation propensity. The top-scoring ABC transporters’ orthologs (*Mus musculus*—mouse ABCB5, *Ailuropoda melanoleuca*—giant panda ABCB6, *Myotis lucifugus*—little brown bat ABCG1 and *Mus musculus* ABCG4) were then expressed in *Saccharomyces cerevisiae* with a combined green fluorescent protein and polyhistidine tag, enabling visualisation and purification. After partial purification and in the presence of the detergent (n-dodecyl-β-D-maltoside), the kinetic parameters of the ATP hydrolysis reactions of the orthologs were determined, as well as the extent of stimulation of their activity when presented with putative substrates. We discuss the efficiency of such bioinformatics approaches and make suggestions for their improvement and wider application in membrane protein-structure determination.

## 1. Introduction

Membrane proteins are one of the largest protein families that interact with, or are a part of, biological membranes. In both prokaryotes and eukaryotes, about 20–30% of all genes encode membrane proteins [1,2,3], whereas approximately 50% of FDA-approved drugs target transmembrane proteins [4]. ATP-binding cassette (ABC) proteins are a class of membrane proteins that will be discussed in this study. ABC proteins form a diverse superfamily, with examples found in all organisms [5]. The majority of ABC protein genes encode membrane-bound or membrane-associated proteins that carry out the transport of different types of molecules across organellar and cytoplasmic membranes [5]. Such ABC transporters can be exporters or importers, with the classification being based on the direction of transport relative to the cytoplasm [6,7]. In addition to import and export, ABC proteins also function as channels and receptors and contribute to the regulation of other biological processes. There are 49 human ABC genes [8]. Based on amino acid homology and domain organisation, these genes may be divided into seven subfamilies (A–G) [8,9].

ABC proteins all contain cytosolic nucleotide-binding domains (NBDs), which bind and hydrolyse ATP, providing energy for transport or other functions. The NBD contains several highly conserved regions, i.e., Walker A and B motifs, H and Q loops, and an ABC signature motif [10]. ABC transporters also have transmembrane domains (TMDs) that are highly variable. The TMDs recognise the substrate and form the route for translocation across the lipid bilayer. All NBDs share the same evolutionary origin and catalytic mechanism, whilst the amino acid sequence of TMDs differs considerably [10]. Human ABC transporters form two categories: (I) full transporters, comprised of two TMDs and two NBDs fused together, and (II) half transporters, which contain one TMD and one NBD fused together. For the latter case, two half transporters are needed to form a functional transporter [11,12,13,14]. ABC proteins are necessary for several processes in the cell, and mutations in their genes contribute to different genetic diseases (see Appendix A Table A1) [9].

The TMCrys server was used in this study to screen about 500 orthologs of the eukaryotic ATP-binding cassette (ABC) family (ABCA-ABCBD and ABCG) [15]. Considering the prediction scoring of TMCrys, four clinically significant ABC proteins were chosen with the aim of biochemical characterisation after protein purification (Table 1). Selected ABC transporters were tagged with green fluorescent protein (GFP) and an octa-His tag and subsequently expressed in *Saccharomyces cerevisiae*. GFP has been widely used as a reporter gene to probe the molecular mechanism or localisation of the protein [16]. Studies comparing the function of the GFP-fused ABC transporter with the non-fused ABC protein showed no effect on their function [17,18,19]. We employed the GFP tag to track the ABC proteins in real time during expression, solubilisation, and purification [20,21]. We observed differences in the expression pattern of the ABC proteins in yeast cells. ABC proteins were solubilised in n-dodecyl-β-D-maltoside (DDM) and purified by nickel-affinity chromatography and then size-exclusion chromatography. We obtained partial purification for three of the four targets and a purification level close to homogeneity for the other target. The effect of potential substrates on the ATPase activity of these ABC transporters was determined.

The principal aim of this study was to produce a protein suitable for crystallisation and/or cryo-electron microscopy. Nuclear magnetic resonance (NMR) has also proven to be a useful alternative for solving structures of small membrane proteins [22,23,24,25]. However, thus far, structures for smaller isolated NBDs of ABC transporters have been published by solution-state NMR [26,27]. The TMCrys scoring system used in this study could also be useful for finding ABC transporter subdomains and other NMR-tractable membrane proteins.

## 2. Materials and Methods

### 2.1. Yeast Cells

*Saccharomyces cerevisiae* FGY217 yeast cells [*MATα ura3-52 lys2*Δ*201 pep4*Δ] were kindly provided by David Drew’s lab at Imperial College London. For this study, yeast glycerol stock stored at −80 °C was used for transformation. FGY217 yeast cells cannot synthesise uracil (URA3 knockout) and have a pep4 deletion, which inhibits the expression of the yeast vacuolar proteinases [28]. As a result, the decreased degradation and improved expression of membrane proteins have been reported in this strain [29]. This yeast cell line has been used to express numerous eukaryotic membrane proteins for purification and crystallisation [30].

### 2.2. Expression Vector

The p424GAL1 expression vector was kindly provided by David Drew’s lab at Imperial College London. For this research work, p424GAL1 plasmid DNA stored at −80 °C was used. This plasmid carries a URA3 gene as a selective marker and the GAL1 promoter (inducible by galactose). There is also a C-terminal tobacco etch virus (TEV) cleavable site—a GFP-fusion tag that is codon-optimised for expression in yeast and is followed by an 8-His tag [30]. The GFP fluorophore has peak excitation at a wavelength of 488 nm and an emission of 512 nm.

### 2.3. TMCrys Screening

TMCrys—version 1.1 is a software tool that predicts the likelihood of membrane protein crystallisation success [15]. A total of 487 orthologs of the ATP-binding cassette family (subfamilies ABCBA to ABCBD and subfamily ABCBG) were screened by TMCrys. Protein sequences were obtained from the Uniprot database and uploaded to the TMCrys server. The output of TMCrys was recorded, which was then evaluated, and orthologs with high crystallisation scores were selected for a protein biochemistry pipeline leading to structural studies.

### 2.4. Constructs

Four ATP-binding cassette genes were synthesised for the following protein targets:

Mouse ABCB5 (mABCB5).
Giant panda ABCB6 (gpABCB6).
Little brown bat ABCG1 (bbABCG1).
Mouse ABCG4 (mABCG4).

ProteoGenix synthesised the constructs (15 rue de la Haye, 67300, Schiltigheim, France). The protein sequences were taken from the Uniprot database, reverse-translated, and codon-optimised for expression in *Saccharomyces cerevisiae* [31]. In all constructs, a yeast-like Kozak sequence, AAAACA, was inserted before the start codon (ATG). The primary structure of the encoded protein was a determining factor in construct design. In ABCB5 and ABCB6, the N-terminus of the fused domains begins with a TMD, with the cytosolic NBD at the C-terminus. GFP, and then the purification tag, were therefore added at the C-terminus. However, for ABCG1 and ABCG4, a TMD is at the C-terminus whilst an NBD is at the N-terminus; hence, the purification tag followed by the GFP was added at the N-terminus. In summary, the mouse ABCB5 gene and giant panda ABCB6 gene were introduced into the p424GAL1 expression vector between 5′ BamH1 and 3′ Xma1/Sma1, downstream to the galactose (GAL1) inducible promoter and upstream to the GFP and the 8His tag. Similarly, the ABCG1 and ABCG4 genes were cloned under the galactose promoter using 5′ BamH1 and 3′ EcoR1 restriction sites. 8His and GFP tags, as well as TEV sites, were added to the ABCG1 and ABCG4 sequences at the N-termini. The TEV cleavage site, GFP, and 8His tags of the p424GAL1 vector were removed by restriction digestion with BamH1 and EcoR1. All sequences of the codon-optimised constructs can be obtained from the corresponding author.

### 2.5. Transformation into Competent E. coli Cells (DH5α)

The transformation of all of the four constructs into highly competent DH5α cells was performed by the heat shock method [32]. Afterwards, a mini preparation of a recombinant protein-containing plasmid was performed using a Qiagen miniprep kit [33].

### 2.6. Transformation into FGY217 Yeast Cells

The transformation of all four constructs into FGY217 yeast cells was carried out using the lithium acetate method [34]. Briefly, FGY217 yeast cells were grown on the YPD agar plates to prepare a yeast culture for transformation. A single colony of the FGY217 yeast strain was grown in 5 mL YPD medium in a shaker-incubator at 250 rpm overnight at 30 °C. The overnight culture was diluted to an OD600 of 0.1 into a pre-warm YPD media and the cells were grown under the same conditions until an OD600 of 0.8–1 was reached. The yeast cells were pelleted by centrifugation at 3000× *g* for 10 min at room temperature, and the cell pellet was resuspended in 1 mL dH_2_O and transferred to a 1.5 mL Eppendorf tube and vortexed briefly. Cells were again centrifuged at maximum speed for 30 s at room temperature, and the cell pellet was resuspended in 1 mL dH_2_O. Moreover, 100 µL is enough for a single transformation, so 100 µL of yeast cells was transferred to a sterile 1.5 mL Eppendorf tube and centrifuged at maximum speed for 30 s at room temperature and the supernatant was discarded. Transformation mixture: For each transformation, the transformation mixture was prepared with 240 µL of PEG 3500 50% (*w*/*v*), 36 µL of 1 M LiAc, 50 µL of freshly prepared 2 mg/mL salmon sperm DNA and 1 µL of 100 ng/µL recombinant plasmid DNA (construct) + 33 µL H_2_O. The yeast pellet was resuspended in the transformation mixture and vortexed followed by heat shock for 25–30 min at 42 °C in a water bath. Cells were then micro-centrifuged at 12,000× *g* for 30 s at room temperature. The supernatant was discarded, and the pellet was resuspended in 400 µL dH_2_O and spread onto on a YNB media plate supplemented with 2% glucose (*w*/*v*) and grown under uracil selection for 3–4 days at 30 °C. Plates were stored at 4 °C for up to one month.

### 2.7. Large Scale/Shake-Flask Cell Culture (12L)

For all four constructs, the same approach was used for large-scale protein expression. One litre of starting culture was prepared by inoculating a lawn of cells or a single colony from the plate into uracil-deficient YNB media. The culture was grown overnight in a shaking incubator at 230 rpm and 30 °C. The following day, twelve 2 L baffled flasks, with each flask containing 1 L of YNB media, were inoculated with 80 mL of starting culture and grown under the same conditions. A spectrophotometer set to 600 nm was used to measure the optical density (OD) of cells periodically. The level of glucose was measured using glucose strips (Medi-test Glucose, Macherey-Nagel, Germany) every 1–2 h. The culture was allowed to grow until the glucose was completely consumed and an OD600 of 0.8–1.0 was reached. Before galactose induction, glucose consumption is essential for protein expression [35]. When glucose was completely consumed and an OD600 of 0.8–1 was reached, protein expression was induced by 2% galactose (*w*/*v*) and 8% glycerol (*v*/*v*), which has previously been proven to boost the expression levels of other ABC proteins [36], and the culture was grown for 16 h under the same conditions. Cells were harvested by centrifugation at 3500× *g*, 4 °C for 10 min. The yeast pellet was resuspended in an ice-cold lysis buffer (0.25 M Tris-HCl (pH 7.8), 0.25 M sucrose, 1 mM EDTA, 2 mM DTT) supplemented with protease inhibitors (0.2 mM AEBSF, 6 μM bestatin, 4 μM chymostatin, 7 μM E-64, 20 μM leupeptin, 15 μM pepstatin A, 1 mM PMSF in dry DMSO plus 0.3 mM benzamidine in dH_2_O). The yeast cell suspension was kept at −80 °C until it was needed for protein purification.

### 2.8. Fluorescence Microscopy

Before target protein purification from yeast, the expression of mABCB5, gpABCB6, bbABCG1, and mABCG4 protein in yeast was confirmed by fluorescence microscopy. For this, 15 µL of harvested yeast cells were mixed with 15 µL of glycerol (50% *v*/*v*). The cells were placed on the glass slide and covered with the coverslip. Cells were analysed by a Zeiss Fluorescence microscope using a 60× objective lens. Images were taken with a Cool snap HQ2 CCD camera (Photo metrics) using Micromanager v1.4.23 software. As all constructs have GFP tag, therefore, set of images was taken at 100–200 m/s using a specific band pass GFP filter setting (Alexa Fluor 488). Image J version 1.52a was used to analyse the images [37,38].

### 2.9. Microsome Preparation

Microsomes were prepared by slightly modifying the bead mill process as described by [36,39]. Frozen yeast cells were thawed on ice. For the breakage of yeast cells, acid-washed glass beads (400–600 μm in diameter) were used in a 1:1 ratio (200 g glass beads: 200 g yeast cells). Bead beating was carried out in the steel chamber of a midi bead beater system (Biospec, Bartlesville, OK, USA)) in a two-minute cycle with a one-minute rest on ice, for a total of 10–12 min of bead beating time. The outer portion of the steel chamber was filled with dry ice during the process to keep the sample as cold as possible. The slurry mixture was allowed to remain on ice for 5 min after bead beating to sediment the glass beads from the slurry. To remove unbroken cells and cellular debris, the suspension was collected carefully and centrifuged at 14,000× *g* for 10 min at 4 °C. To collect the microsomes (whole inner membrane fraction), the supernatant was centrifuged at 120,000× *g* for 90 min at 4 °C. The supernatant was then discarded, and the microsome pellet was resuspended in high-salt buffer (50 mM Tris-HCl (pH 7.8) (Fischer Scientific, Loughborough, UK), 500 mM NaCl (Fischer Scientific, Loughborough, UK), 10% *v*/*v* glycerol (Acros organics, Geel, Antwerpen, Belgium) with the help of a glass homogeniser and centrifuged 100,000× *g* for 45 min. The microsome pellet was resuspended in solubilisation buffer with no detergent (50 mM Tris-HCl (pH 8), 150 mM NaCl, 10% *v*/*v* glycerol), and total protein concentration was measured by Bradford assay. In-gel GFP scanning following electrophoresis and Western blotting with an anti-His antibody was used to verify the presence of the protein of interest in microsomes. The microsome suspension was flash-frozen in liquid nitrogen and kept at −80 °C until used.

#### 2.9.1. Microsome Solubilisation

For all four proteins, the microsome solubilisation step was standardised based on the work of Jackson et al. 2018b [40]. Microsomes were solubilised with final protein concentrations at 2.5 mg/mL. To solubilise gpABCB6 microsomes, a stock solution of 10% DDM (*w*/*v*) in solubilisation buffer was made and mixed with microsomes to a final concentration of 2.5 mg/mL total protein and 2% DDM (*w*/*v*). For mABCB5, bbABCG1 and mABCG4 microsomes, 2% DDM: 0.02% cholesteryl hemisuccinate (CHS) was used. Solubilisation was performed at 4 °C with gentle end-to-end rotation for 2 h. The suspension was centrifuged at 100,000× *g* for 45 min at 4 °C, and the supernatant containing the solubilised microsomes was collected. The solubilised microsomes can be applied directly to a Ni-NTA affinity column for affinity purification or flash frozen and kept at −80 °C for long-term storage.

#### 2.9.2. Immobilised Metal Affinity Chromatography

The solubilised protein was purified using Ni-NTA chromatography followed by the size-exclusion chromatography [39]. A GE healthcare AKTA FPLC system (Cytiva, Marlborough, MA, USA) with a Frac950 fraction (Cytiva, Marlborough, MA, USA)collection system was used to purify mABCB5, bbABCG1, and mABCG4 using a 5 ml HiFliQ Ni-NTA column (Generon, Slough, Buckinghamshire, UK). Solubilised microsomes were applied to the 5 mL pre-equilibrated Ni-Affinity column with a flow rate of 0.5 mL/min. Flow-through was collected in a falcon tube for further analysis. To eliminate loosely bound contaminants, the column was washed with 10 column volumes (CV) of 20–30 mM imidazole elution buffer B (50 mM Tris-HCl (pH 8), 150 mM NaCl, 10% glycerol, 0.1% DDM, 0.01% CHS, 1 mM β-mercaptoethanol, 0–500 mM imidazole) (wash 1) and then 8CV 50–80 mM imidazole elution buffer B (wash 2). At 200–250 mM imidazole concentration, the purified protein was eluted. The column was washed with 5CV of 500 mM elution buffer B to remove all bound proteins.

Ni-NTA loose resin (Qiagen, Manchester, UK) was used to purify gpABCB6. The column was filled with 10 mL Ni-NTA loose resin and equilibrated with purification buffer A. Solubilised microsomes were applied to the Ni-resin, and the column was incubated at 4 °C for two hours with end-to-end rotation. Flow-through was collected by gravity flow. The column was washed with 10 CV of 20 mM imidazole in elution buffer B (wash 1) and 5 CV of 40 mM imidazole in elution buffer B (wash 2). The specifically bound protein was eluted using 150 mM imidazole in elution buffer B. The column was washed with 5 CV of 500 mM imidazole in elution buffer B to remove all bound protein from the Ni-resin. The corresponding fractions were run on SDS-PAGE and analysed for GFP fluorescence before colloidal Coomassie staining. The elution fractions containing purified protein were pooled. Imidazole-free purification buffer was used to dilute the 150–200 mM elution fraction, bringing the imidazole concentration down to less than 5 mM. Using Millipore20 concentrators (MercK, Darmstadt, Germany) with a 100 kDa cut-off, the diluted fraction of approximately 100 mL was then concentrated to a final volume of 1–2 mL for size-exclusion chromatography or until the absorption of GFP was observable by eye (a greenish yellow colour) in the concentrator. Concentrated protein was split into 500 μL aliquots and snap-frozen for size-exclusion chromatography.

#### 2.9.3. Size-Exclusion Chromatography (SEC)

A GE healthcare AKTA FPLC system with a Frac900 collecting system was used to perform size-exclusion chromatography. The column (SepFast™ 6–6000 kDa, Generon Slough, Buckinghamshire, UK) was equilibrated with 2 CV of degassed ultrapure water followed by a 1.5 CV of degassed SEC buffer (50 mM Tris-HCl (pH 8), 4% glycerol, 0.1% DDM, 0.01% CHS). The sample was ultra-centrifuged at 100,000× *g* at 4 °C for 15 min to remove aggregates and then injected into a 500 μL super loop, prior to being loaded onto the equilibrated column. Chromatography was carried out at a flow rate of 0.5 mL/min, with 1 mL fractions collected for 1.2 CV. Absorbance at 280 nm (UV280) was recorded to assess total proteins, and GFP fluorescence was recorded to follow the target protein. SDS-PAGE was used to confirm the location of the target protein in eluted fractions. Fractions enriched in the target protein were pooled and concentrated with a 100 kDa cut-off (Millipore) (MercK, Darmstadt, Germany) filter before being aliquoted and snap-frozen for later use.

### 2.10. Protein Characterisation

#### 2.10.1. ATPase Activity (Chifflet Assay)

This experiment employed a standard classical molybdate method for inorganic phosphate determination [41], and the ATPase activity of samples was determined using Na-ATP in the presence of DDM (a mild detergent) and Mg^2+^. In the first row of a 96-well plate, phosphate standards with concentrations of 0, 1, 2, 3, 4, 5, 6, 8, 10, 12, 16, and 20 nmol were set up to plot a standard curve. In the second row, 10 µg of purified protein was added to each well to obtain a good signal. For substrate-stimulated ATPase activity, 10 µM substrate dissolved in dimethyl sulfoxide (DMSO) was added to each well, with a final concentration of 0.1% *v*/*v* DMSO. Glutathione was used at 5 mM concentration with no DMSO. The reaction was started by adding ATP. Moreover, 5 mM ATP stock (adenosine 5′-triphosphate disodium salt) was prepared in ATPase buffer [50 mM Tris-HCl (pH 7.4), 150 mM NH_4_Cl, 5 mM Mg_2_SO_4_]. The plate was incubated at 25 °C for 25 min. Afterwards, in each well 40 µL of buffer A [12% (*w*/*v*) SDS] was added to stop the ATPase reaction by protein denaturation. This was followed by 100 µL of buffer F [Freshly prepared (1:1 mixture of buffer B (1% (*w*/*v*) ammonium molybdate) and buffer E (6% (*w*/*v*) ascorbate in 1 M HCl)] and incubated at room temperature for 5 min.

Following incubation, 100 µL of buffer D [2% (*w*/*v*) sodium citrate, 2% (*w*/*v*) sodium metaarsenite, 1% (*v*/*v*) acetic acid in dH_2_O] was added to the plate, and the plate was incubated for 15 min at 37 °C. The absorbance of the sample was measured in a spectrophotometer at 800 nm. The phosphate standard was plotted, and the ATPase activity of the purified protein was calculated using the phosphate standard as a reference point. Substrate-stimulated ATPase activity was calculated at a fixed concentration of 3 mM ATP at 25 °C for 25 min. Kinetics data were fitted using GraphPad prism version 7.04 (Dotmatics, Boston, MA, USA) according to the Michaelis–Menten equation [42,43]. The ATPase activity was tested in 3–4 independent experiments; therefore, data are shown as mean ± SD. A one-way ANOVA test followed by Dunnett’s test was applied to examine the statistical significance in GraphPad Prism 7. Maximal activity (Vmax) and a Michaelis–Menten constant (Km) were determined by Michaelis–Menten kinetics fittings.

#### 2.10.2. Dynamic Light Scattering

Protein samples were diluted to 1 mg/mL in buffer (100 mM Tris pH 8, 10% glycerol, 150 mM NaCl, 0.1% DDM, and 0.02% CHS). Each experimental solution, which contained 10 µg protein, buffer, and additional additives such as 10 µM cyclosporin A, was made up in a final volume of 10 µL and incubated on ice for 15 min. The experiment was carried out with the UNcle spectroscopic apparatus, and 9 µL of each solution was loaded in a 16-well UNcle capillary cassette. The dynamic light scattering (DLS) was recorded at 20 °C. The duration of data gathering was set to 5 s.

#### 2.10.3. Membrane Thermal Shift Assay

A total reaction of 80 µL of solubilised microsomes (5 mg/mL) was set up. Solubilised microsomes were heated in a PCR machine at various temperatures (20–90 °C) for 3 min to establish a thermal denaturation curve. After heating, solubilised microsomes were ultra-centrifuged at 100,000× *g* for 20 min at 4 °C to precipitate the denatured protein during the heating process. The supernatant was collected and run on 8% polyacrylamide gel, scanned for GFP fluorescence, and subjected to Western blot analysis. To determine the relative thermal stabilisation of ABCG4 by tyrosine kinase inhibitors (TKIs), solubilised microsomes were first incubated with 10 µM of each TKI dissolved in dimethyl sulfoxide (DMSO) for 60 min at 37 °C and then heated at 70 °C for 3 min and ultra-centrifuged. Then, the supernatant was subjected to Western blot analysis. TKIs were incubated with ABCG4 solubilised microsomes for 60 min at 37 °C, which provided sufficient time and an appropriate temperature for interactions [44]. The hypothesis was that, in the presence of TKIs, ABCG4 would be more stable at 70 °C and be less prone to unfold and precipitate. The immunoblot data were analysed by Image J software, and the graph was plotted using GraphPad Prism 7.

### 2.11. Homology Modelling

MODELLER 9.24 (Slilab, University of California, San Francisco, CA, USA) was used for the homology modelling of mABCG4 protein [45]. For the selection of the templates, a PSI-BLAST search against Protein Data Bank (PDB) was performed. The PDB file of each template was downloaded from PDB. Basic python scripts were downloaded from the tutorial and modified according to the target protein. The multiple alignment and alignment of the target protein sequence to the template sequence was performed using the align command in MODELLER. The model of a target sequence was generated based on the alignment against the multiple templates using the “model-mult.py” command and the model was evaluated by Discrete Optimised Potential Energy (DOPE) score using the “evaluate-model.py” routine. A dimeric mABCG4 model was built in using an experimentally determined structure of ABCG2 (PDB ID: 6VXF) in dimeric form. One copy of the ABCG4 monomer model was superimposed onto chain A of 6VXF and a second copy of the monomer was superimposed onto chain B of 6VXF; then, both were combined using the “combine” command into a single model and saved as a PDB file.

## 3. Results

### 3.1. Expression in Yeast

**Figure 1:**
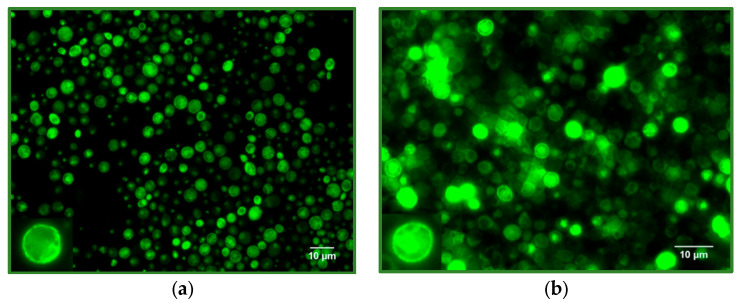

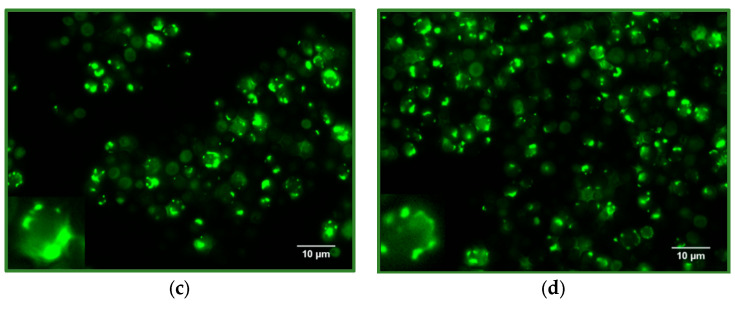
Fluorescence microscopy using the Alexa Fluor 488 filter to detect the GFP tag (green fluorescence). Yeast cells at 16 h post induction are shown expressing (**a**) mouse ATP-binding cassette protein subfamily B member 5 (mABCB5); (**b**) giant panda ATP-binding cassette protein subfamily B member 6 (gpABCB6); (**c**) little brown bat ATP-binding cassette protein subfamily G member 1 (bbABCG1) and (**d**) mouse ATP-binding cassette protein subfamily G member 4 (mABCG4). Bottom left inset in each panel shows a magnified image of a representative yeast cell from the sample.

**Figure 2:**
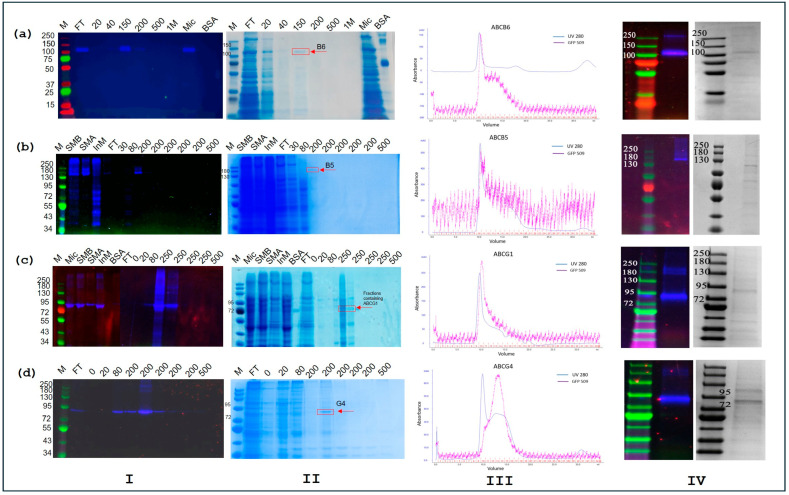
Protein purification via a two-step method. (**a**) Purification of gpABCB6; (**b**) Purification of mABCB5; (**c**) Purification of bbABCG1; (**d**) Purification of mABCG4. For each protein Column I shows Ni-NTA purification fractions on SDS PAGE scanned for GFP-fluorescence. Column II shows the corresponding gel after Coomassie-staining. Column III shows size-exclusion chromatograms with the darker line showing the GFP fluorescence intensity. Column IV shows SDS PAGE of the pooled size-exclusion fractions containing the target protein after concentration. Key: M: protein marker in kDa; FT: Flow through; Mic: Microsomes; BSA: Bovine serum albumin SMB: Solubilised microsomes before centrifugation SMA: Solubilised microsomes after centrifugation InM: Insoluble microsomes, Numbers: indicate imidazole concentration in purification buffer in millimolar (mM).

The expression of mABCB5, gpABCB6, bbABCG1, and mABCG4 proteins in yeast was confirmed by fluorescence microscopy (Figure 1). For mABCB5 and gpABCB6, much of the GFP fluorescence was found around the periphery of the cell (Figure 1a,b), as might be expected for a plasma membrane protein [46]. However, some GFP fluorescence was detected internal to the cell, which may be due to mis-targeting to other membranes or the cleavage of the expressed proteins and/or GFP tag by cellular proteases (see later). In contrast, bbABCG1 and mABCG4 microscopy images displayed a more punctate GFP signal, located just inside the plasma membrane (Figure 1c,d). These microscopy data were interpreted as suggesting that bbABCG1 and mABCG4 were targeted to another yeast compartment. Although the formation of ABCG1 and ABCG4 protein aggregates could also explain the punctate localisation, the ability to solubilise these proteins with a mild detergent (see later—Figure 2) argues against this interpretation. For each protein, the time course of expression was also investigated using fluorescence microscopy and the quantitation of the GFP signal. All four proteins showed a maximal expression in the 14 to 22 h time window, which was followed by a slow loss of expression up to the end of the experiment (60 h timepoint). For subsequent experiments, larger-scale expression aimed at protein yield was halted within the 14–22 h time window by harvesting the cells and storage at −80 °C.

### 3.2. Protein Purification via Two Step Method

Figure 2 shows representative data illustrating purification attempts for the four proteins. Each row represents progress for one of the four proteins from left to right, beginning with SDS-PAGE data for Ni-NTA affinity chromatography. This is followed by 280 nm absorbance and GFP fluorescence data for SEC elution and, lastly, SDS-PAGE for pooled and concentrated SEC fractions.

#### 3.2.1. gpABCB6

A single band with a molecular weight of roughly 120 kDa was present on SDS-PAGE using the GFP fluorescence for detection. No indication of SDS-stable dimers or oligomers nor lower-mass cleavage products were found for this protein. The 120 kDa band coincided with expectations for gpABCB6-GFP (gpABCB6 = 92 kDa + the additional cleavable GFP and affinity tag = 21 kDa). Comparisons of the GFP band intensities in the 150 mM elution fraction, the (unbound) flow-through and the microsomes suggested good protein binding to the column and a high solubilisation efficiency (Figure 2a). The monitoring of protein elution from the SEC column indicated protein eluting around the exclusion limit of the column as well as some lower-molecular-mass proteins. From the GFP detection trace, it was concluded that gpABCB6-GFP eluted from the column either as a narrow high-molecular-mass peak, or as a broader shoulder that was expected to contain ABCB6 dimers. SDS-PAGE of the pooled and concentrated SEC fractions showed ABCB6 as the most prominent band, but several weaker bands showed purification was only partially achieved. These are likely to be yeast Ni-binding proteins including some with intrinsic poly-His sequences that have approximately the same mass as gpABCB6 [47].

#### 3.2.2. mABCB5

This protein eluted from the Ni-NTA column at about 150 mM imidazole, viewed with the GFP detection as a single band of roughly 150 kDa (Figure 2b). This is likely to be the expressed mABCB5-GFP construct (137.4 kDa + the GFP and affinity tag = 21 kDa). Comparison of the band intensities on the gel using GFP detection indicated the high protein retention of mABCB5-GFP on the affinity column, although solubilisation efficiency with DDM was about 50%. However, it was clear from the SDS-PAGE experiments that the materials not solubilised by DDM were the (undesirable) higher-molecular-mass aggregates and cleavage products (only C-terminal cleavage products are detected in this experiment). Subsequent purification of mABCB5-GFP by SEC was carried out, yielding a similar profile to gpABCB6-GFP above (i.e., with evidence of some protein aggregation). The amount of mABCB5-GFP eluting from the column was lower than expected, perhaps as a consequence of non-specific binding to the column resin. As a result, the purification of mABCB5-GFP was worse than for gpABCB6-GFP, with several other protein bands more abundant than the mABCB5-GFP band on SDS PAGE gels stained with Coomassie.

#### 3.2.3. bbABCG1

A fluorescent band on the gels with a molecular weight of ~94 kDa was readily detected, and it most likely corresponds to GFP-bbABCG1 (sequence weight 75.4 kDa + the additional cleavable N-terminal GFP and affinity tag = 21 kDa). Comparison of the GFP band intensity of the various fractions indicated that solubilisation efficiency with DDM was rather lower for bbABCG1, although on the positive side, the insoluble material was again enriched in (undesirable) SDS-resistant aggregates (Figure 2c). A very high efficiency of protein binding to the affinity column was indicated for bbABCG1 although elution at 250 mM imidazole was effective still. Interestingly, the high protein loading for the 250 mM imidazole fractions indicates some evidence for N-terminal cleavage products that elute somewhat earlier from the column, whilst SDS-resistant aggregates emerge later. The SEC of Ni-NTA-enriched GFP-bbABCG1 displayed profiles that indicated unexpectedly high mass distribution, and SDS-PAGE also showed the formation of higher-mass SDS-resistant aggregates of the protein, although the monomer band was still the most abundant band on the Coomassie-stained gels. These purification data imply that GFP-bbABCG1 may be unstable after the removal of imidazole and concentration.

#### 3.2.4. mABCG4

A fluorescent band at the ~93 KDa band was likely to be the GFP-mABCG4 construct (mABCG4 = 72 kDa + the additional cleavable GFP and affinity tag = 21 kDa). When comparing the amount of protein in the 200 mM fraction with the amount in the flow-through, Ni-NTA affinity chromatography showed a protein binding of about 80–90%, and this efficiency remained consistent in each purification run (Figure 2d). Some evidence of SDS-resistant higher-mass oligomers was indicated for the elution fraction with the higher protein concentration, although no evidence for N-terminal cleavage fragments was observed. In contrast to the other three proteins, GFP-mABCG4 showed an encouraging symmetrical SEC elution profile, with only a minor shoulder at the exclusion limit of the column. Purity was also better for ABCG4, with Coomassie staining showing only one significant additional band at around 100 kDa. Interestingly, when detected using an anti-His tag antibody, DDM-purified GFP-mABCG4 also displays an additional band at 100 kDa on Western blots (see later, Section 3.5). This band does not give rise to GFP fluorescence (Figure 2d, IV); hence, it seems possible that it could arise from a sub-population of GFP-mABCG4 where the GFP has failed to fold and the chromophore has failed to form. Folding efficiency for GFP has been reported to be around 70%, although in yeast this value may be different [48,49].

### 3.3. ATPase Activity Measurements

**Figure 3:**
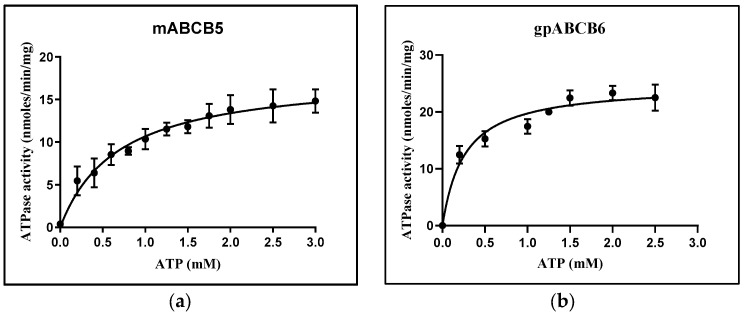

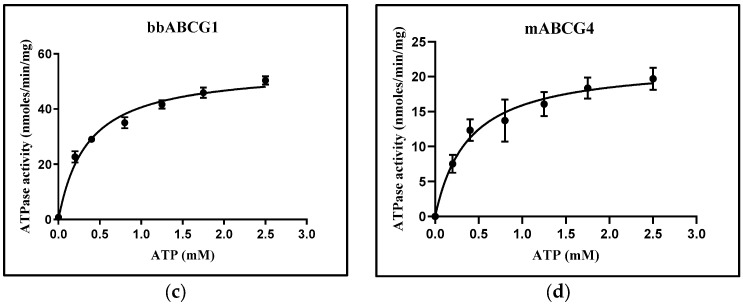
ATPase activity measured by Chifflet assay. (**a**) Apo-mABCB5 in detergent (DDM) shows a maximal ATPase activity of 18.0 ± 0.9 nmol/min/mg and a Km estimated at 0.77 ± 0.10 mM. (**b**) Apo-gpABC6 in detergent (DDM) shows a maximal ATPase activity of 24.8 ± 0.9 nmol/min/mg and a Km estimated at 0.26 ± 0.03 mM. (**c**) Apo-bbABCG1 in detergent (DDM) shows a maximal ATPase activity of 54.8 ± 1.6 nmol/min/mg and a Km estimated at 0.35 ± 0.03 mM. (**d**) Apo-mABCG4 in detergent (DDM) shows a maximal ATPase activity of 21.7 ± 1.2 nmol/min/mg and a Km estimated at 0.40 ± 0.07 mM.

The basal- and substrate-stimulated ATPase activities of partially purified ABC proteins were measured using the Chifflet assay [41]. As at least three of the proteins tested had significant degrees of contamination, there was a strong likelihood of detecting the ATPase activity of contaminating proteins. All four protein fractions displayed saturable ATPase activity (Figure 3), with values for the Michaelis constant (Km) that were similar to other reports [19,50,51,52]. Unstimulated specific activity for all four protein fractions were below 100 nmol/min/mg protein.

#### 3.3.1. mABCB5

**Figure 4:**
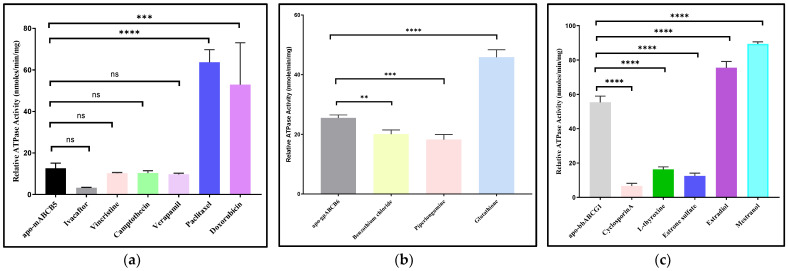

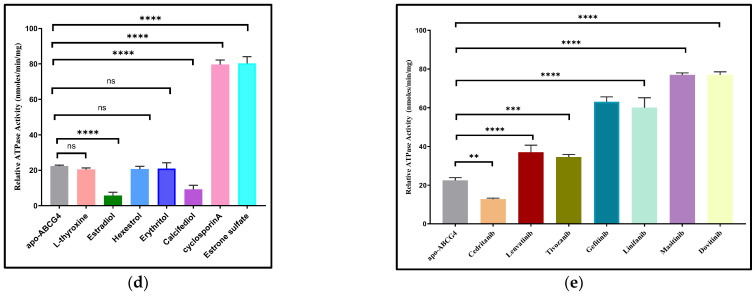
Relative ATPase activities in a detergent environment. (**a**) Relative maximal ATPase activities of mABCB5 showed that paclitaxel stimulated ATPase activity increased by 5-fold compared to that of the basal activity. (**b**) Relative maximal ATPase activities of gpABCB6 showed that glutathione stimulated ATPase activity increased by 2- fold compared to that of the basal activity. (**c**) Relative maximal ATPase activities of bbABCG1 showed that mestranol stimulated ATPase activity increased by 6-fold compared to that of the basal activity. (**d**) Relative maximal ATPase activities of mABCG4 showed that cyclosporinA and estrone stimulated ATPase activity increased by 4-fold compared to that of the basal activity. (**e**) Relative maximal ATPase activities of mABCG4 in the presence of tyrosine kinase inhibitors. Each data point represents the mean ± SD calculated from three independent experiments. When there is no visible error bar, the SD is less than the corresponding symbol. A one-way ANOVA test is applied for comparison of statistical significance. The *p*-values <0.01, 0.001 and 0.0001 are indicated with **, ***, and **** compared to apo-mABCG4. ns: non-significant.

Human ABCB5 substrates stimulated the ATPase activity of partially purified mABCB5 fractions. Doxorubicin and paclitaxel, known substrates for human ABCB5 [53,54], stimulated the ATPase activity of mABCB5-GFP fractions roughly four-fold; however, camptothecin, the (lower) toxicity of which was strongly correlated with ABCB5 expression [53], did not stimulate ATPase activity (Figure 4a). Verapamil, which may inhibit the ABCB5 efflux of doxorubicin in human cells, did not affect the ATPase activity of the partially purified material. Kawanobe et al., reported a basal ATPase rate of 65 nmol/min/mg and Km 1.8 mM for ABCB5-containing vesicles obtained from insect cells overexpressing the protein using a baculovirus system [54]. This specific activity is somewhat higher than the activity reported here, although again these were preparations where ABCB5 would be present with many other ATPases. Kawanobe and co-workers found 1.5-fold and 3-fold increases in ABCB5 basal ATPase activity in the presence of doxorubicin and docetaxel, respectively [54].

#### 3.3.2. gpABCB6

Partially purified gpABCB6 exhibited basal ATPase activity with a Vmax of about 25 nmol/min/mg and a Km of 0.3 mM. This is consistent with recent reports for human ABCB6 Vmax 27 nmol/min/mg [55] and similar to the detergent-purified basal ATPase activity of human ABCB6 [56]. The substrate stimulation of ATPase activity was observed with glutathione (two-fold stimulation), which has been implicated as a co-factor for ABCB6 transport [57], but no stimulation was detected for piperlongumine and benzothium chloride, which were reported to be capable of inhibiting porphyrin accumulation that was being driven by human ABCB6 transport activity (Figure 4b) [58].

#### 3.3.3. bbABCG1

Partially purified bbABCG1 exhibited basal ATPase activity with Vmax of 55 nmol/min/mg and a Km of 0.35 mM. Kalpana and co-workers reported a Vmax of detergent-purified *Arabidopsis thaliana* ABCG1 of 19 nmol/min/mg and Km 1.8 mM [59]; however, amino acid sequences of plant ABCG members differ significantly from mammals [60]. Hirayama and co-workers reported no activity for detergent-purified ABCG1 [19], but this could be because of a different expression system. Substrate stimulation by estradiol and its synthetic derivative mestranol increased the bbABCG1 ATPase activity by 1.3-fold and 1.5-fold, respectively, whilst the charged estrone sulfate showed some inhibitory effect on the ATPase activity. Cyclosporin A and L-throxine, likely inhibitors of ABCG1 transport, led to lower rates of ATP hydrolysis (Figure 4c) [61].

#### 3.3.4. mABCG4

mABCG4 exhibited ATPase activity with a Vmax of 22 nmol/min/mg and a Km of 0.39 mM, respectively. This is in accord with the 18 nmol/min/mg Vmax reported for ABCG4 overexpressed in insect vesicles [61]. In contrast to bbABCG1, the stimulation of mABCG4 ATPase activity occurred with cyclosporin A and estrone sulfate, with the former result being unexpected as cyclosporin A is frequently found to inhibit ABC transporter transport function. L-thyroxine, hexestrol, erythritol, estradiol, and calcifediol did not show any significant effect on the mABCG4 ATPase activity (Figure 4d). mABCG4 ATPase activity was also tested in the presence of tyrosine kinase inhibitors (TKIs), potentially transported substrates of this protein [62,63,64]. The data showed that except for cediranib, the six TKIs (dovitinib, gefitinib, lenvatinib, tivozanib, masitinib, and linfanib) stimulated the mABCG4 ATPase activity by 1.5- to 3-fold, suggesting that they may be substrates for this murine version of the protein and implying some substrate overlap with ABCG2 (Figure 4e) [44].

### 3.4. Dynamic Light Scattering Data for mABCG4

**Figure A1:**
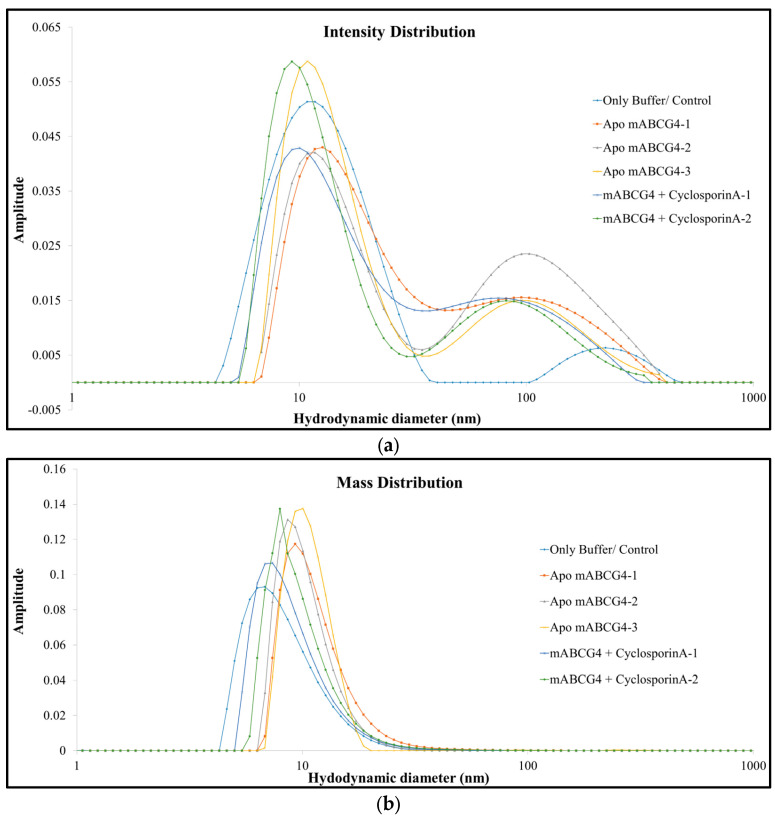
Dynamic light scattering (DLS) profiles of mABCG4 and in the presence of cyclosporin A. Buffer only control show particles (detergent micelles) of about 7 nm diameter. (**a**) A bimodal intensity distribution is displayed for the samples in the intensity distribution plot, with distinct populations with hydrodynamic diameters ~7–13 nm and >50 nm. The latter likely represent aggregates which contribute disproportionately to the intensity profile. The mass distribution plot (**b**) compensates for this over-emphasis of the scattering of large particles within the sample.

**Figure A2:**
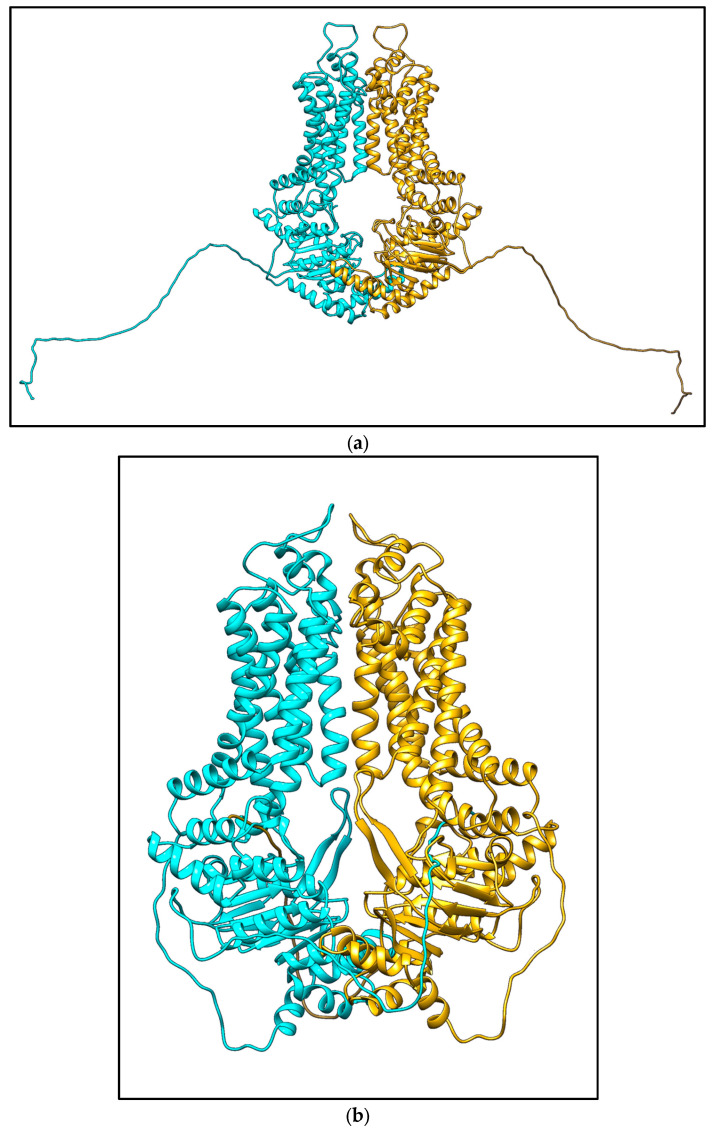
mABCG4 protein structure. Panel (**a**) shows dimeric model of mABCG4 protein determined in Chimera. Panel (**b**) shows alpha fold mABCG4 dimer structure. RMSD between MODELLER and AlphaFold mABCG4 protein structure is 1.134 Å determined in Chimera.

We performed dynamic light scattering (DLS) to determine the homogeneity, aggregation, and size distribution of purified mABCG4 protein (see Appendix A Figure A1). Buffer control samples displayed DLS consistent with a homogeneous population of particles of about 6–7 nm diameter (panel b), with a tiny proportion of particles being >100 nm. This likely corresponds to the properties of the DDM detergent micelles. Purified mABCG4 intensity distribution graphs demonstrated a bimodal distribution of particles. The first peak showed a particle hydrodynamic diameter of ~11–13 nm, in agreement with predictions for the mABCG4 homology model (13.4 nm, Appendix A Figure A2). The second broad peak in the DLS profiles corresponded to particles of diameter ~50 to ~150 nm, with a mode of around 95 nm. These larger particles likely correspond to aggregates of mABCG4. Because these aggregates individually scatter light much more than the 10 nm diameter particles, the relative abundance of the 10 nm particles is still considerably greater than the protein aggregates [65], as demonstrated in the mass distribution plots. The polydispersity index of the apo-mABCG4 was 0.2. Further DLS experiments with cyclosporin A were carried out on two separately purified batches of mABCG4; in each case, the mass distribution plots indicated a skew to a slightly smaller diameter. Whether this shift was due to minor structural changes to the protein upon ligand binding was not clear, but this merits further study. Other experiments were carried out with nucleotide addition, but these showed inconsistency between protein batches and occasional large increases in hydrodynamic diameter, suggestive of the formation of large aggregates.

### 3.5. Thermal Shift Stability Data for mABCG4

**Figure 5:**
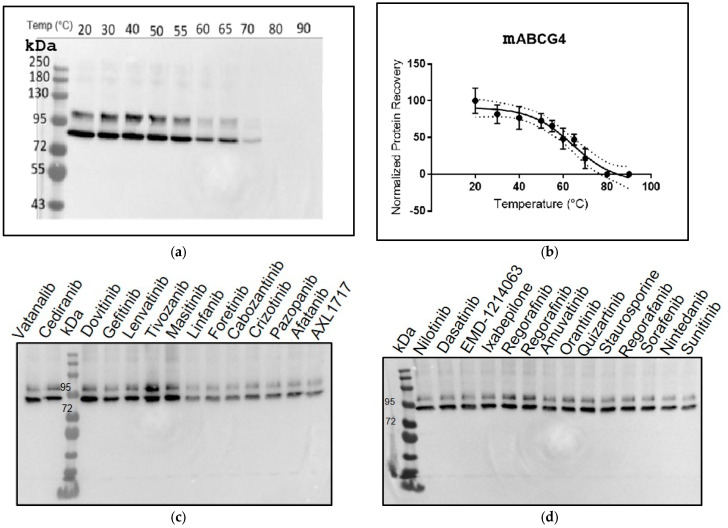
Membrane thermal shift assay of mABCG4. DDM-solubilised mABCG4-containing microsomes were incubated at each temperature for 3 min. (**a**) the Western blot of mABCG4 which disappeared at 70 °C; (**b**) the graphical representation of quantification of protein recovery analysed by ImageJ after thermal unfolding; (**c**,**d**) Western blots of GFP-mABCG4 incubated in the presence of 10 tyrosine kinase inhibitors (TKIs) at 20 °C and then heated to 70 °C prior to centrifugation and loading on the gel.

In order to investigate the possible effects of tyrosine kinase inhibitors (TKIs) on GFP-mABCG4, we employed a thermal shift assay, which has previously been employed for ABCG2 as well as other ABC transporters [44,66] to detect the binding of substrates via shifts in the thermal stability of the target protein. Figure 5 shows the thermal stability of ABCG4 at elevated temperatures. Unfolding of the protein leads to aggregation and loss of the protein band on SDS-PAGE gels (as detected by Western blotting). GFP-mABCG4 incubated at room temperature runs as two bands—a major one at the expected mass of about 90 kDa and a minor band at about 100 kDa. As discussed above, this latter band may represent a population of GFP-mABCG4 in yeast where the GFP has failed to fold. Both bands disappear concurrently as the incubation temperature is increased, with a mid-point for denaturation of around 60 °C, whilst at 80 °C, nearly all the GFP-mABCG4 has disappeared from the gel. We therefore screened a collection of TKIs for their ability to bind to GFP-mABCG4, as detected by an increase in thermostability at 70 °C. Figure 5c,d shows the results of this screen. Several TKIs appeared to increase the thermostability of ABCG4 at 70 °C, with significant overlap with the data displayed in Figure 4. Dovitinib, tivozanib and masitinib appear to be the most obvious stabilising TKIs in this screen, and these compounds also stimulated the ATPase activity of the GFP-mABCG4 fractions (Figure 4e).

## 4. Discussion

The aim of this study was to increase the chance of the successful expression, purification and biochemical characterisation (including structure determination) of target membrane proteins by utilising bioinformatics tools. We utilised software that has been developed for identifying membrane proteins likely to be tractable for structure determination. The software calculates the probability of success of the solubilisation, purification, and crystallisation of membrane proteins based on their amino acid sequence, as well as predicting the success of all three steps combined [15]. Rather than restricting the scoring to human ABC transporters, we also included mammalian orthologs since utilising orthologs of human membrane proteins has been a successful strategy in membrane protein-structure determination [67]. After initiating the study, developments in cryo-electron microscopy (cryo-EM) emerged that allowed high-resolution structure determination for many more ABC transporters than was previously possible by crystallography. Nevertheless, two of the three factors scored (solubilisation and purification) by TMCrys are still highly relevant for cryo-EM studies. We chose four high-scoring ABC transporter orthologs with >70% sequence similarity to the human protein version where structural information was (at the time) lacking. Currently, only one of the four targets has no structure deposited (ABCG4). Nevertheless, this study has allowed us to test a rational strategy for selecting targets for biochemical studies.

For expressing the targets, we utilised a *Saccharomyces cerevisiae* expression system for membrane proteins developed by Drew and colleagues [46]. This system incorporates GFP into the recombinant protein, which facilitates the tracking of the protein temporally, spatially and during purification. The fluorescence microscopy data shown in Figure 1 indicated more plasma membrane-located GFP fluorescence in mABCB5- and gpABCB6-expressing yeast cells. In contrast, bbABCG1- and mABCG4-expressing yeast cells displayed punctate GFP fluorescence. Whether these differences relate to the way yeast targets membrane proteins to the plasma membrane is not clear; however, the relative success in the purification of ABCG1 and particularly ABCG4 versus ABCB5 and ABCB6 implies that punctate localisation is not a strong indicator of misfolding and aggregation. Similarly, the purification gels argue against the notion that (cleaved) N-terminal GFP-containing fragments of ABCG1 and ABCG4 may persist with punctate localisation after synthesis and degradation at the endoplasmic reticulum stage [68,69,70]. For ABCB5 and ABCB6, a C-terminal tag would require the complete synthesis/folding of the preceding protein before GFP would appear, and, therefore, these recombinant proteins may reflect populations that have already escaped the ER quality control. It would be interesting to see the precise intracellular localisation either by the use of confocal microscopy or by the use of antibodies to different organellar marker proteins [71].

Despite the assistance from bioinformatics, the success rate for the purification of the four membrane protein targets was still low (25%). However, the first stages in the procedure, (overexpression and protein solubilisation with a mild and commonly used detergent—DDM) was surprisingly efficient (100% success for at least 50% solubilisation of the four targets). Furthermore, the SDS-PAGE results for some of the targets imply that incomplete solubilisation of membrane-bound protein by DDM can be beneficial in weeding out aggregated and/or cleaved protein fragments. The high success rate for solubilisation we encountered may have been fortuitous given the small sample size, but it merits further monitoring. Similarly, all the target proteins bound efficiently to the Ni-NTA column (as judged by comparison with the flow-through fraction), and three were readily eluted by the addition of 200 mM imidazole (ABCB5 being the probable exception). Attrition appeared to be more significant at the second stage of purification, especially for ABCB5, and it was at this stage that aggregation and polydispersity became apparent for three of the four targets. The various hurdles encountered in this study will be familiar to many membrane protein biochemists and prior to structural studies, much effort is usually invested into obtaining good Gaussian elution profiles for target proteins on SEC (such as those shown for mGFP-ABCG4 in this report). The ‘ortholog approach’ pioneered by Gouaux and co-workers and the ‘mutagenesis for thermal stability’ approach developed intensively by Tate and co-workers are examples of such exhaustive strategies for membrane protein-structure determination [67,72]. Unsurprisingly, many membrane protein studies cannot progress much further than using enriched membranes. Hence, a bioinformatics-aided approach merits consideration for biochemical studies, and the development of better prediction tools for successful purification is suggested.

Knowledge of the range of substrates that ABC transporters transport is often difficult to obtain. One explanation is that some ABC transporters are able to transport several structurally unrelated substrates, and it is likely that the four targets chosen here fall into this category. Although three of the four targets failed to be purified, the stimulation of the ATP hydrolysis activity of ABC transporters by the addition of transported substrates allows some indication of the likely substrate range for these orthologs [73,74,75,76]. Although all the partially purified protein fractions had relatively low specific ATPase activities in the absence of substrates, the results obtained were consistent with studies for other eukaryotic ABC transporters that have been studied in detergents, including ABCB1, ABCB6, ABCC7, and ABCG2 [52,55,77,78,79]. Furthermore, the low purity of three fractions would be consistent with lower ATPase activity. The significant stimulation of the ATPase activity of all the final fractions was a good indication that the partially purified proteins were able to maintain activity in the dodecyl maltoside micelles. For ABCB5, which was the worst in terms of purity, the ATPase activity of ABCB5 increased by 3-fold and 5-fold in the presence of doxorubicin and paclitaxel, respectively. For ABCB6, glutathione caused the ATPase activity of ABCB6 to increase three times, which is consistent with an earlier report [55]. ABCG1 is involved in the translocation of lipids and sterols [80,81]. For ABCG1, estradiol and mestranol stimulated ATPase activity, but cyclosporin A, L-thyroxine, and estrone had an inhibitory effect, consistent with earlier studies [61].

The GFP-mABCG4 construct purified the best of the targets, and for this protein, there appeared to be some overlap with human ABCG2 substrates despite only 29% amino acid sequence identity between murine ABCG4 and human ABCG2. Estrone sulfate stimulated the ATPase activity of mABCG4 approximately four-fold. The binding of cyclosporin to ABCG4 may be expected, but its stimulation of ATPase activity was unexpected. Protein kinase inhibitors have recently been identified as substrates for ABCB1 (P-glycoprotein) and ABCG2 [44,82]. For ABCG4, we found a roughly three-fold stimulatory effect of dovitinib, gefitinib, lenvatinib and masitinib.

In conclusion, of the four choices that were guided by bioinformatics, mABCG4 appeared to be the best target for cryo-EM studies. The protein has ATPase activity that can be stimulated by a sterol that is likely to be a transported substrate (estrone sulfate), and it also appears to bind and be stabilised by a series of tyrosine kinase inhibitors that are anti-cancer drugs. These drugs also stimulate the ATPase activity of ABCG4. However, in our hands, ABCG4 has so far proven intractable for cryo-EM, largely because there was contamination with free detergent micelles. It seems likely that this problem will be resolved, but it illustrates, again, the peculiar difficulties in obtaining the structures of membrane proteins.

A future avenue to solve the structure of ABC transporters is purification using a copolymer SMA (polystyrene-co-maleic acid) rather than detergents [83]. SMA solubilises the membrane into tiny discs of lipid bilayers surrounded by a polymer, known as SMALPs (SMA lipid particles). ABC transporters encapsulated in SMALPs can be purified by affinity chromatography and then subjected to cryo-EM to determine their structure. Compared to detergent solubilisation, there is high level of purity and improved stability [84]. SMALP-encapsulated ABC transporters are able to bind ligands comparably with those in native membranes or detergent micelles. ATPase assays can be employed on purified ABC proteins to find substrates that modulate the ABC transporter activity (inhibitors or activators); this is based on the substrates’ potential to increase the ATPase activity [85]. Large chemical libraries can be screened at high throughput using membrane vesicles or purified protein [86].

## Figures and Tables

**Table 1:** TMCrys scores of selected orthologs. Protein sequences were obtained from Uniprot database. The probability of success at each stage is estimated. A score of 1 is the maximum and 0 is the minimum score.

Organism	Solubilisation	Purification	Crystallisation	Reliability of Prediction
mABCB5	0.698	0.973	0.591	0.856
gpABCB6	0.613	0.925	0.847	0.864
bbABCG1	0.724	0.975	0.897	0.878
mABCG4	0.720	0.930	0.920	0.857

## Data Availability

The raw data supporting the conclusions of this article will be made available by the authors on request.

## Appendix A

**Table A1:** Importance of ABC family. Diseases marked with * are reported on gene card [87]. Other diseases are taken from Uniprot database [88].

Protein	A.A	Mol.wt (kDa)	Functions	Diseases
ABCA1	2261	254.3	Cholesterol efflux onto HDL	High-density lipoprotein deficiency 1 (HDLD1) High-density lipoprotein deficiency 2 (HDLD2)
ABCA2	2436	269.8	Macrophage lipid metabolism and neural development.	Intellectual Developmental Disorder with Poor Growth With or without seizures * Acoustic neuroma *
ABCA3	1704	191.4	Multidrug resistance	Pulmonary surfactant metabolism dysfunction 3 (SMDP3)
ABCA4	2273	255.9	N-retinylidene-phosphatidylethanolamine (PE) efflux	Stargardt disease (STGD1) Retinitis pigmentosa 19 (RP19) Age-related macular degeneration Fundus flavimaculatus (FFM) Cone-rod dystrophy 3 (CORD3)
ABCA5	1642	186.5	Urinary diagnostic marker for prostatic intraepithelial neoplasia (PIN)	Hypertrichosis, Congenital generalized with or without Gingival hyperplasia * Lysosomal diseases *
ABCA6	1617	184.3	Macrophage lipid homoeostasis	Autosomal recessive congenital icthyosis *
ABCA7	2146	234.4	Cholesterol efflux	Alzheimer disease 9 (AD9)
ABCA8	1581	179.3	Transports certain lipophilic drugs	Autosomal recessive congenital icthyosis *
ABCA9	1624	184.4	Might play a role in monocyte differentiation and macrophage lipid homeostasis	N/A
ABCA10	1543	175.8	Cholesterol-responsive gene	Donnai-Barrow syndrome (DBS) *
ABCA12	2595	293.2	Might involve in lipid homeostasis and also has implications for prenatal diagnosis	Ichthyosis, congenital, autosomal recessive 4A (ARCI4A) Ichthyosis, congenital, autosomal recessive 4B (ARCI4B)
ABCA13	5058	576.2	Inherited disorder affecting the pancreas	Schizophrenia * Stargardt disease *
ABCB1	1280	141.5	Multidrug resistance	Inflammatory bowel disease 13 (IBD13) Colchicine resistance *
ABCB2/TAP1	808	87.2	Peptide transport	Bare lymphocyte syndrome 1 (BLS1)
ABCB3/TAP2	703	75.6	Peptide transport	Bare lymphocyte syndrome 1 (BLS1)
ABCB4	1279	141.5	Phosphatidylcholine (PC) transport	Cholestasis, progressive familial intrahepatic, 3 (PFIC3) Cholestasis of pregnancy, intrahepatic 3 (ICP3) Gallbladder disease 1 (GBD1)
ABCB5	812	138.6	Melanogenesis	Borna disease * Melanoma *
ABCB6	842	93.8	Iron transport/Heme synthesis	Dyschromatosis Universalis Heredetaria (DUH) Microphthalmia Familial pseudo hyperkalemia (FPH)
ABCB7	753	82.6	Fe/S cluster transport	Anemia, sideroblastic, spinocerebellar ataxia (ASAT)
ABCB8	718	70.9	Intracellular peptide trafficking across membranes	Abcd syndrome * Anemia, sideroblastic, spinocerebellar ataxia *
ABCB9	766	84.5	Located in lysosomes	Bile acid synthesis defect, Congenital, 5 * Bare lymphocyte syndrome 1 *
ABCB10	738	79.2	Export of peptides derived from proteolysis of inner-membrane proteins/Heme biosynthesis	Developmental coordination disorder Stereotypic movement disorder
ABCB11	1321	146.4	Bile salt transport	Progressive familial intrahepatic cholestasis
ABCC1	1531	171.6	Drug resistance	Dubin–Johnson syndrome * Pseudoxanthoma elasticum *
ABCC2	1545	174.2	Organic anion efflux	Dubin–Johnson syndrome Bilirubin metabolic disorder *
ABCC3	1527	169.3	transport of biliary and intestinal excretion of organic anions	Dubin–Johnson syndrome * Extrahepatic syndrome *
ABCC4	1325	149.5	Nucleoside transport	Biliary tract disease * Dubin-Johnson syndrome *
ABCC5	1437	160.6	Nucleoside transport	Primary Angle-closure glaucoma * Episodic kinesigenic Dyskinesia 1 *
ABCC6	1503	164.9	active transport of drugs directly or indirectly,	Pseudoxanthoma elasticum (PXE) Arterial calcification of infancy
ABCC7/CFTR	1480	168.5	Chloride ion channel (same as CFTR gene in cystic fibrosis)	Cystic fibrosis Congenital bilateral absence of vas deferens (CBAVD)
ABCC8	1581	176.9	Sulfonylurea receptor	Familial hyperinsulinemic hypoglycaemia 1 (HHF1) Leucine-induced hypoglycaemia (LIH) Transient neonatal diabetes mellitus Permanent neonatal diabetes mellitus
ABCC9	1549	174.2	Encodes the regulatory SUR2A subunit of the cardiac K^+^(ATP) channel	Cardiomyopathy, dilated 1O (CMD1O) Atrial fibrillation, familial, 12 (ATFB12) Hypertricotic osteochondrodysplasia (HTOCD)
ABCC10	1464	161.6	Multidrug resistance	Borna disease * Abcd syndrome *
ABCC11	1382	154.3	Drug resistance in breast cancer	Apocrine gland secretion variation in * Lateral sinus thrombosis *
ABCC12	1359	152.3	Multidrug resistance	Episodic Kinesigenic dyskinesia 1 * Dubin-Johnson syndrome *
ABCD1	745	82.9	Very-long-chain fatty acid (VLCFA) transport	Adrenoleukodystrophy (ALD) Hypoadrenocorticism, Familial *
ABCD2	740	83.2	Major modifier locus for clinical diversity in X-linked ALD (X-ALD)	Adrenoleukodystrophy (ALD) Zellweger syndrome
ABCD3	659	75.5	Involved in import of fatty acids and/or fatty acyl-coenzyme As into the peroxisome	Congenital bile acid synthesis defect 5 (CBAS5)
ABCD4	606	68.6	Lysosomal transporter	Methylmalonic aciduria and homocystinuria type cbIJ (MAHCJ)
ABCG1	678	75.6	Cholesterol transport	Tangier disease * Sitosterolaemia *
ABCG2	655	72.3	Toxicant efflux, drug resistance	Gout/Hyperuricemia
ABCG4	646	71.9	Macrophage lipid homeostasis	Abcd syndrome * Sitosterolemia *
ABCG5	651	72.5	Sterol transport	Sitosterolemia 2 (STSL2)
ABCG8	673	75.7	Sterol transport	Gallbladder disease4 (GB4) Sitosterolemia 1 (STSL1)
